# Identification of candidate genes that regulate the trade-off between seedling cold tolerance and fruit quality in melon (*Cucumis melo* L.)

**DOI:** 10.1093/hr/uhad093

**Published:** 2023-05-09

**Authors:** Lili Li, Qiong Li, Bin Chen, Jiyu Wang, Fei Ding, Panqiao Wang, Xiuyue Zhang, Juan Hou, Renren Luo, Xiang Li, Jingwen Zheng, Sen Yang, Luming Yang, Lei Zhu, Shouru Sun, Changsheng Ma, Qin Li, Ying Li, Jianbin Hu

**Affiliations:** College of Horticulture, Henan Agricultural University, Zhengzhou 450002, China; College of Horticulture, Henan Agricultural University, Zhengzhou 450002, China; College of Horticulture, Henan Agricultural University, Zhengzhou 450002, China; College of Horticulture, Henan Agricultural University, Zhengzhou 450002, China; College of Horticulture, Henan Agricultural University, Zhengzhou 450002, China; College of Horticulture, Henan Agricultural University, Zhengzhou 450002, China; College of Horticulture, Henan Agricultural University, Zhengzhou 450002, China; College of Horticulture, Henan Agricultural University, Zhengzhou 450002, China; College of Horticulture, Henan Agricultural University, Zhengzhou 450002, China; College of Horticulture, Henan Agricultural University, Zhengzhou 450002, China; College of Horticulture, Henan Agricultural University, Zhengzhou 450002, China; College of Horticulture, Henan Agricultural University, Zhengzhou 450002, China; College of Horticulture, Henan Agricultural University, Zhengzhou 450002, China; College of Horticulture, Henan Agricultural University, Zhengzhou 450002, China; College of Horticulture, Henan Agricultural University, Zhengzhou 450002, China; College of Horticulture, Henan Agricultural University, Zhengzhou 450002, China; The Seed Management Station of Zhengzhou City, Zhengzhou 450001, China; College of Horticulture, Henan Agricultural University, Zhengzhou 450002, China; College of Horticulture, Henan Agricultural University, Zhengzhou 450002, China

## Abstract

Trade-offs between survival and growth are widely observed in plants. Melon is an annual, trailing herb that produces economically valuable fruits that are traditionally cultivated in early spring in China. Melon seedlings are sensitive to low temperatures, and thus usually suffer from cold stress during the early growth period. However, little is known about the mechanism behind the trade-offs between seedling cold tolerance and fruit quality in melon. In this study, a total of 31 primary metabolites were detected from the mature fruits of eight melon lines that differ with respect to seedling cold tolerance; these included 12 amino acids, 10 organic acids, and 9 soluble sugars. Our results showed that concentrations of most of the primary metabolites in the cold-resistant melons were generally lower than in the cold-sensitive melons; the greatest difference in metabolite levels was observed between the cold-resistant line H581 and the moderately cold-resistant line HH09. The metabolite and transcriptome data for these two lines were then subjected to weighted correlation network analysis, resulting in the identification of five key candidate genes underlying the balancing between seedling cold tolerance and fruit quality. Among these genes, *CmEAF7* might play multiple roles in regulating chloroplast development, photosynthesis, and the ABA pathway. Furthermore, multi-method functional analysis showed that *CmEAF7* can certainly improve both seedling cold tolerance and fruit quality in melon. Our study identified an agriculturally important gene, *CmEAF7*, and provides a new insight into breeding methods to develop melon cultivars with seedling cold tolerance and high fruit quality.

## Introduction

Plants are constantly exposed to a variety of adverse conditions during growth, including biotic and abiotic stresses. Faced with the trade-off between survival and growth, plants have evolved complex regulatory mechanisms to respond to stresses, but these often cause adverse effects on plant growth and development as well as on quality and yield [1–3]. Trade-offs between the two functions (growth and defense) may result from at least three non-exclusive mechanisms: (1) traits may be directly limited by resource competition (allocative costs); (2) traits may be genetically related through pleiotropic or linkage imbalances (genetic costs); or (3) coexpression of traits may be penalized by the plant environment (energy constraints or ecological costs) [2]. It is widely known that commercial cultivars with high quality and yield mostly lack adequate tolerance to environmental stresses. Therefore, exploring the genes that balance crop quality and stress not only contributes to our understanding of the trade-off mechanism but also favors the development of strategies to breed crop cultivars that are both high-quality and stress-resistant. There is accumulating evidence to show that some genes and microRNAs are involved in the growth-defense trade-off without obvious penalties on plant growth and yield [4–6], and some even show increased yield [4, 6–9]. However, no research has been reported in horticultural crops to date, especially for cucurbits.

Cold stress is one of the most widespread abiotic stresses that affects the normal growth and development of crops, and cold can cause irreversible damage to yield and quality [10, 11]. Melon (*Cucumis melo* L.) originated in Africa and has been domesticated across the whole of Asia, particularly in India [12, 13]. As a typical thermophilic crop, melon plants are sensitive to low temperatures throughout the growth period, especially at the seedling stage [14, 15]. In China, the world's largest producer of melon fruits, melon cultivation is usually advanced to early spring to ensure maximum benefit to the farmers; however, there is always the risk that low temperatures in early spring (e.g. commonly occurring late spring cold periods) will delay plant growth, thus decreasing fruit quality and yield [16, 17]. The current extended-season melon cultivars grown in China have not been bred to have high levels of cold tolerance. Therefore, the exploration of cold tolerance genes can provide valuable genetic resources for the breeding of cold-tolerant melon varieties. Compared with model plants such as *Arabidopsis thaliana*, rice, and tomato, research into cold tolerance in melon lags behind. CMCT505_Chr.1 was identified as a key locus that controls cold tolerance in melon in a genome-wide association study (GWAS), but it was not genetically dissected [18]. At present, no QTL or gene related to low temperature tolerance has been cloned using forward genetics in melon. Also, only a few low-temperature-related QTLs have been mapped in other cucurbits, such as cucumber [19, 20] and pumpkin [21]. Recently, two transcription factors, an abscisic acid-responsive element (ABRE)-binding factor 1 (*CmABF1*) and a C-repeat binding factor 4 (*CmCBF4*) were reported to bind directly to the promotor of *CmADC*, a gene that encodes a key synthetase for putrescine biosynthesis, enhancing cold tolerance in melon seedlings [22].

Tolerance to environmental stresses and high quality of harvested organs has long been considered to be two important goals in horticultural breeding; however, in most cases, there is a contradiction between the two traits. In fact, plant breeding is the process that breeders use to coordinate the trade-offs between different agronomic traits. Therefore, characterizing the genes that balance quality and stress could be extremely valuable in breeding high-quality and stress-resistant horticultural cultivars. A recent report described a new function of the classic cold-responsive gene *OsDREB1C*; plants overexpressing (OE) *OsDREB1C* had 41.3% to 68.3% higher yields than did the wild-type (WT) plants due to increased grain number per panicle that resulted from activating transcription of genes related to carbon assimilation, nitrogen uptake and transport, and flowering [23]. Similar cases were also found with the genes *ZmDRESH8* and *ZmMYBR38,* which could improve both seedling drought resistance and yield in maize [24]. However, no information is available about the trade-offs between seedling cold tolerance and fruit quality in melon. In this study, a comparative analysis was performed in eight melon lines with contrasting cold resistance using a multi-omics method, and five genes (*MELO3C016749.2*, *MELO3C012147.2* (*CmEAF7*), *MELO3C026791.2*, *MELO3C010343.2*, and *MELO3C024337.2*) were identified as key candidates to balance seedling cold tolerance and fruit quality. Transient expression assay results indicated that *CmEAF7*, which has a splice donor variant in line H581, could enhance seedling cold tolerance and fruit quality (Primary metabolite) in melon. Our study provides new gene resources for improving cold tolerance and fruit quality in melon (primary metabolite), as well as helping to describe the mechanism that underlies these two important traits.

## Results

### Identification of cold resistance in melon seedlings

**Figure 1:**
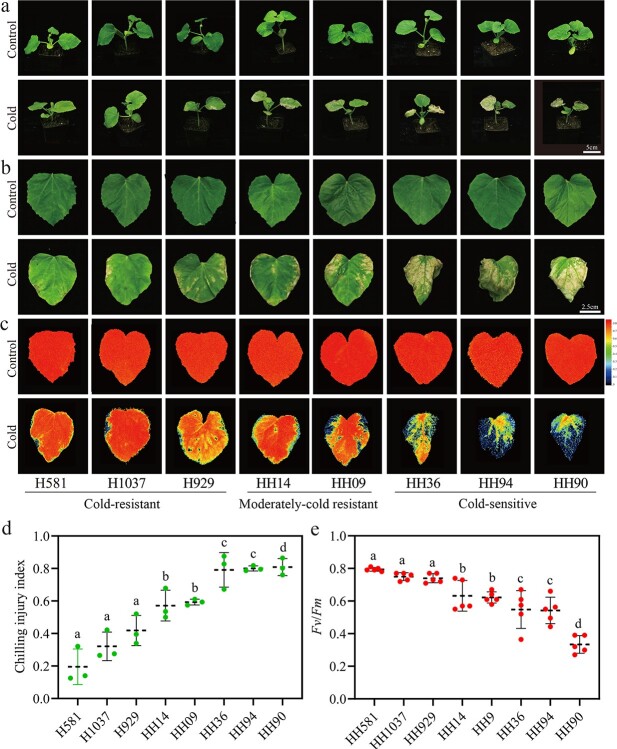
The cold tolerance of eight inbred lines of melon. (a-b) Cold response phenotypes in seedlings (a) and leaves (b). (c) Chlorophyll fluorescence images of the first true leaves. (d) Chilling injury index. There were three biological replicates for each line with 15 seedlings in each replicate. (e) Chlorophyll fluorescence (*Fv*/*Fm*), n = 5. Values are means ± SD. Statistical difference were determined by one-way analysis of variance (ANOVA). The same letter above a column indicates no significant difference at *p* < 0.05.

Cold tolerance in the eight melon genotypes was evaluated by examining the cold-injury phenotypes (Figure 1a and b) and the chlorophyll fluorescence (Figure 1c) of seedling leaves (Figure 1); the two methods directly reflect the level of damage to tissues of the seedling leaves. Based on the degree of damage to the leaves, the eight genotypes could be divided into three groups; (1) a cold-resistant group (H581, H1037, and H929), (2) a moderately cold-resistant group (HH14 and HH09), and (3) a cold-sensitive group (HH36, HH94, and HH90). Also, we calculated the CII value and the maximum photosynthetic efficiency (*Fv*/*Fm*) for each of the eight genotypes; the cold-resistance group had low CII values of <0.4 and a high *Fv*/*Fm* value of >0.7, the moderately cold-resistant group had medium CII values of 0.5 to 0.6 and *Fv*/*Fm* values of 0.6 to 0.7, and the cold-sensitive group had high CII values of >0.8 and low *Fv*/*Fm* values of <0.6 (Figure 1d and e).

**Figure 2:**
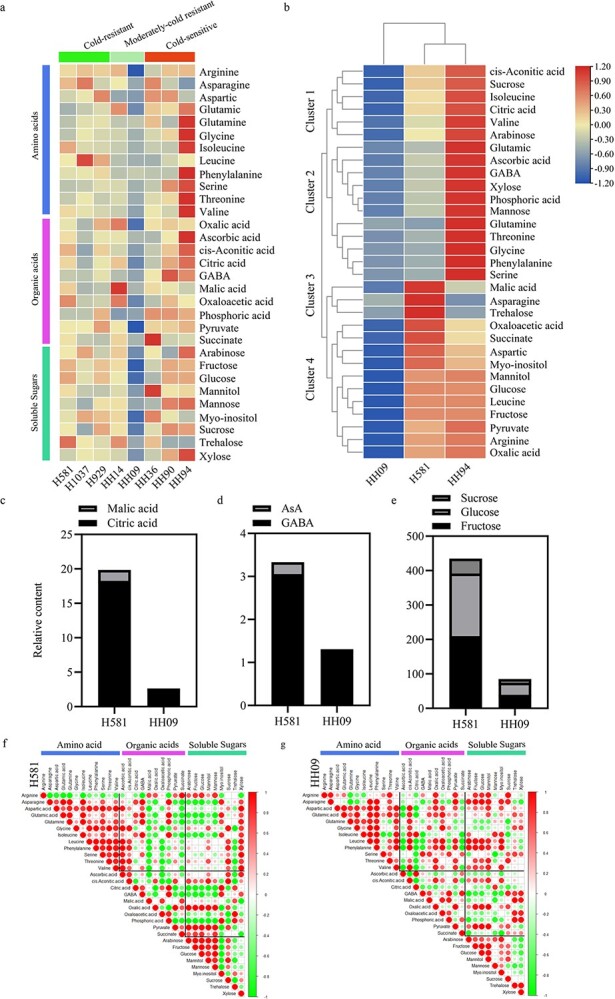
Metabolite analysis was performed by gas chromatography–mass spectrometry (GC–MS) in extracts of melon fruit flesh. (a) Heat map of metabolite levels in fresh fruits from eight inbred melon lines. There were four biological replicates for each inbred line. (b) Comparison of metabolite levels in inbred lines H581, HH94, and HH09. (c–e) The contents of organic acids, AsA, GABA, and soluble sugars in H581 and HH09. (f–g) Visualization of metabolite-metabolite correlations in melon lines H581(f) and HH09 (g).

**Figure 3:**
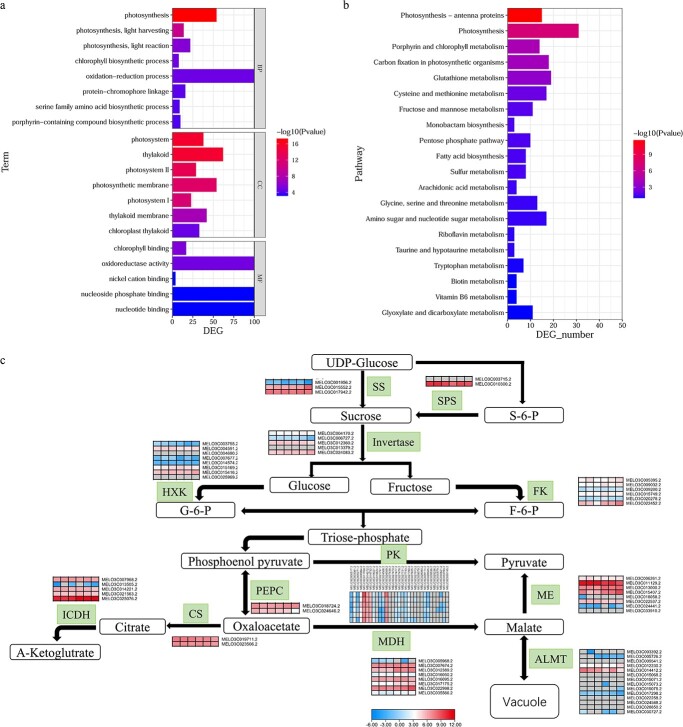
Gene expression analysis in H581 and HH09 fruits. (a and b) GO and KEGG gene enrichment analysis of differentially expressed genes (DEGs). The three main GO categories (biological process, cellular component, and molecular function) are indicated on the right side of the figure in (a). (c) The expression levels of genes involved in the biosynthesis of organic acids and sugars. There were three biological replicates for each line. Left represent H581 and right represent HH09. Grids represent the expression levels of genes, which are shown as FPKM values.

### Analysis of primary metabolites in melon flesh from fruits of inbreds in the different cold-tolerant groups

Metabolic profiles of mature fruit flesh from the eight melon lines that differ with respect to cold tolerance were performed using GC–MS. A total of 31 primary metabolites were detected by 48 standards, including 12 amino acids, 10 organic acids, and 9 soluble sugars (Figure 2a). Overall, the metabolites in the cold-sensitive melon were more abundant than those in cold-tolerant melon. Typically, the contents of metabolites were the highest in the cold-sensitive line HH94, followed by the cold-tolerant line H581 and the moderately cold-tolerant line HH09 (Figure 2a). PCA revealed that fructose showed the largest difference within the 31 metabolites in the eight melon genotypes, followed by glucose, sucrose, pyruvate, and citric acid (Figure S1a and b). Moreover, HH09, H581, and HH94 were distributed in different quadrants, indicating that they had large differences in metabolite contents (Figure S1a).

We performed cluster analysis on the metabolites from HH09, H581, and HH94, and they were divided into four clusters based on the differences in metabolite levels (Figure 2b). In cluster 1, the contents of important soluble sugars and organic acids, such as sucrose and citric acid, were lowest in HH09, higher in H581, and highest in HH94. In cluster 2, the contents of some important metabolites (glutamic acid, AsA, and GABA) in HH09 were lower than that in H581, but were highest in the cold-sensitive inbred line HH94; The contents of several amino acids including glutamine, threonine, glycine, phenylalanine, and serine were similar in HH09 and H581, but were much lower than in HH94. In cluster 3, the contents of malic acid, asparagine, and trehalose were higher in H581 than in HH09 and HH94. In cluster 4, some important primary metabolites (aspartic acid, leucine, arginine, myo-inositol, glucose, fructose, oxalic acid, and pyruvate) were higher in both H581 and HH94 than in HH09 (Figure 2b). In conclusion, the cold-resistant melon line H581 and the cold-sensitive melon line HH94 accumulated higher levels of some primary metabolites in fruits than did the moderately cold-resistant melon line HH09 (Figure 2a and b).

A further comparison of the important sugars (sucrose, glucose, and fructose) and organic acids (malic acid and citric acid) that determine fruit sweetness, as well as AsA and GABA, which are very important to human health, were conducted in the two lines H581 and HH09 that differ in their cold tolerance. We found that the concentrations of the above substances in fruits were significantly higher in H581 than in HH09 (Figure 2c, d, and e). We next performed correlation analysis of 31 primary metabolites in H581 and HH09 fruits (Figure 2f, g) and extreme differences emerged between the soluble sugars and amino acids, organic acids, and amino acids in the two lines (Figure 2f, g). The analysis showed that the differences between soluble sugars and amino acids were negatively correlated in H581, but positively correlated in HH09 for fructose, glucose, mannose, mannitol, and trehalose. Similarly, the correlations between organic acids and amino acid metabolites were also significantly different between H581 and HH09. Among them, malic acid, oxalic acid, oxaloacetic acid, and pyruvate were significantly negatively correlated with many amino acids in H581, but they were positively correlated with these same amino acids in HH09. In addition, citric acid, an important organic acid, was positively correlated with asparagine, glutamic acid, glutamine, and isoleucine in H581, but only positively correlated with isoleucine and glutamine, and negatively correlated with the other 10 amino acids in HH09. Ascorbic acid, which is an important antioxidant, was positively correlated with 11 amino acids, but not with arginine, in H581, whereas AsA was negatively correlated with all 12 amino acids in HH09. GABA showed a similar pattern; it was positively correlated with 11 amino acids but not arginine in H581 and negatively correlated with seven amino acids in flesh of HH09 fruits.

Furthermore, it is worth mentioning that these two inbred lines also had some differences in the correlations between the levels of amino acids and amino acids, soluble sugars and soluble sugars, organic acids and organic acids, and soluble sugars and organic acids (Figure 2f, g). In H581, arginine was negatively correlated with the levels of the other 11 amino acids, while the levels of the other amino acids were positively correlated with one another. Arginine, aspartic acid, serine, and threonine were negatively correlated with three, five, four, and three amino acids in HH09, respectively. Moreover, the comparisons of soluble sugars with soluble sugars showed that the correlations of myo-inositol and mannose levels with those of other sugars were significantly different in H581 and HH09. In the comparisons of organic acids with organic acids, citric acid was significantly positively correlated with GABA, oxaloacetic acid, and phosphoric acid levels in H581, while citric acid was significantly positively correlated with AsA in HH09. In addition, the correlations between citric acid and GABA, malic acid, and GABA, and citric acid and phosphoric acid were opposite in the two inbred lines. In the comparisons of soluble sugars and organic acids, we found that the correlations of inositol and sucrose with organic acids, respectively, AsA, citric acid, malic acid, and succinic with soluble sugars, respectively, were found to be more different in H581 and HH09. In conclusion, the cold-resistant melon line H581 and the moderately cold-resistant line HH09 show significant differences in the contents of primary metabolites, and they show the largest differences in the correlations between the contents of soluble sugars and amino acids, and between organic acids and amino acids.

### Expression analysis of genes involved in soluble sugar and organic acid metabolic pathways in H581 and HH09

A total of 2154 DEGs were obtained from RNA-seq analysis of H581 and HH09 fruit pulp. GO and KEGG pathway enrichment analyses were subsequently performed for these genes (Figure 3 a and b). GO enrichment analysis showed that the DEGs were significantly enriched in biological processes, such as photosynthesis, light harvesting, and reaction in photosynthesis, chlorophyll biosynthetic process, and oxidation–reduction process, among which the significance was the highest for photosynthesis, and the largest number of DEGs were enriched in oxidation–reduction processes. In addition, consistent with the biological process, the proteins encoded by these DEGs are mainly located in the photosystem and thylakoid, and have functions such as chlorophyll binding and oxidoreductase activity (Figure 3a). In addition, KEGG pathway analysis showed that the DEGs were significantly enriched in photosynthesis-antenna proteins, photosynthesis, porphyrin and chlorophyll metabolism, carbon fixation in photosynthetic organisms, and some amino acids, sugars, and organic acids metabolic processes (Figure 3b).

**Figure 4:**
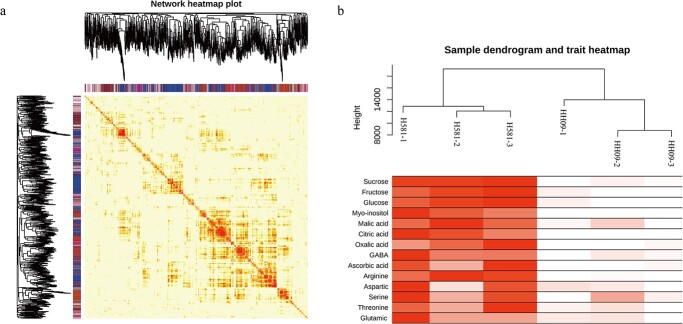
Construction of co-expression regulatory networks in the cold-resistant melon lines H581 and HH09. (a) The cluster dendrogram and network heatmap of genes involved in coexpression module calculation. (b) Sample dendrogram and module trait heatmap for the inbred lines H518 and HH09.

**Figure 5:**
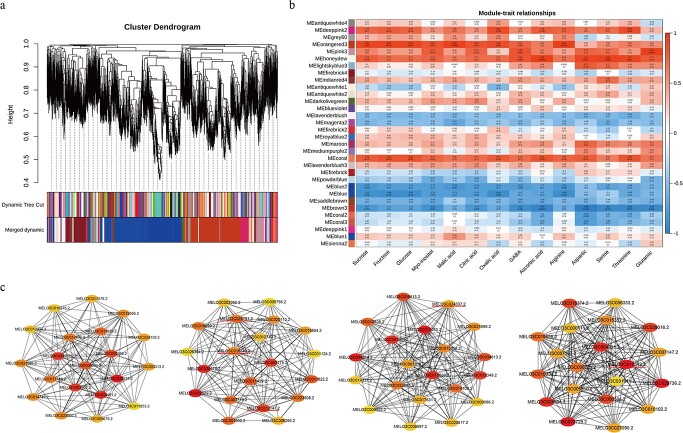
Gene networks and key candidate genes involved in cold tolerance and primary metabolite trade-offs in melon as identified by WGCNA. (a) Clustering dendrogram of the gene coexpression modules. (b) Module-trait relationships based on Pearson correlation coefficients. (c) Gene networks for four modules; from left to right MEbrown3, MEhoneydew, MEorangered3, and MEpink3 (*r^2^* > 0.90, *p* < 0.01).

Moreover, the expression levels of genes that encode key enzymes involved in the metabolism of sugars and organic acids were also quite different in H581 and HH09 (Figure 3c). *MELO3C010300.2*, which encodes sucrose phosphate synthase (SPS), was highly expressed in the fruits of both lines, but its expression level was higher in H581 than in HH09. Hexokinase (HXK) is an important enzyme in glucose metabolism, among which the genes *MELO3C003755.2, MELO3C004591.2,* and *MELO3C007677.2* had higher expression levels in H581 compared to HH09. In contrast, the relative expression of genes for invertase (*MELO3C004170.2*) and fructokinase (FK) (*MELO3C005395.2*, *MELO3C009032.2*, *MELO3C015749.2*, and *MELO3C022452.2*) were lower in H581 than in HH09. Similarly, some genes that encode isocitric dehydrogenase (ICDH) (*MELO3C025076.2*), pyruvate kinase (PK) (*MELO3C003381.2*, *MELO3C005271.*2, *MELO3C011769.2*, *MELO3C021797.2*, *MELO3C024508.2*, *MELO3C027760.2*, and *MELO3C028114.2*), and malate dehydrogenase (MDH) (*MELO3C012389.2*, *MELO3C016095.2*, *MELO3C017175.2*, and *MELO3C035566.2*) in organic acid metabolism also showed lower expression levels in H581 than in HH09. Furthermore, malic acid, an important organic acid in fruits, can be converted to pyruvate through the action of malate enzyme (ME) or stored in vacuoles by malate transporters. In H581, the expression of *MELO3C011129.2* was the highest of eight malate enzyme (ME) genes, and its expression was higher than in HH09. On the contrary, the expression of other ME-encoding genes (*MELO3C013000.2*, *MELO3C015407.2*, and *MELO3C024441.2*) was lower than in HH09. The malate transporter protein (ALMT) family consists of 14 homologous genes in melon, but most of these genes were not expressed in the two inbred lines. In particular, *MELO3C014412.2* had the highest relative expression of all 14 ALMT genes, and it was expressed at a higher level in H581 than in HH09, indicating that malate transport was more active in H581 fruits.

### Weighted gene coexpression network analysis (WGCNA) of primary metabolism in H581 and HH09 fruits

A weighted gene coexpression network analysis (WGCNA) was performed using FPKM values from the transcriptome data, and 32 gene modules were obtained based on the coexpression patterns of the genes. The clustergram and network heatmap were then used to visualize these gene modules, and each gene module was tagged with a unique color (Figure 4a and Figure 5a). For further module-trait correlations, a total of 14 important primary metabolites including sucrose, fructose, glucose, myo-inositol, malic acid, citric acid, oxalic acid, GABA, ascorbic acid, arginine, aspartic acid, serine, threonine, and glutamic acid were selected for clustering and heatmap display in H581 and HH09 (Figure 4b). There is no doubt that the contents of these substances were all higher in fruits of H581 than in HH09 fruits (Figure 4b).

An association analysis was performed between the 32 modules and 14 metabolites, and the results showed that five and two modules were significantly positively and negatively correlated with metabolites, respectively, with correlation coefficients (*r^2^* > 0.80 and *p*-values <0.05) (Figure 5b). Among them, four modules (MEbrown3, MEhoneydew, MEorangered3, and MEpink3) had the highest correlations with metabolites (*r^2^* > 0.90, *p* < 0.01) (Figure 5b and c). In particular, MEbrown3 was significantly negatively correlated with myo-inositol (*r^2^* = 0.81, *p* = 0.05), GABA (*r^2^* = 0.87, *p* = 0.02), AsA (*r^2^* = 0.93, *p* = 0.007), aspartic acid (*r^2^* = 0.99, *p* = 0.0001), serine (*r^2^* = 0.90, *p* = 0.01), threonine (r*r^2^* = 0.89, *p* = 0.02), and glutamic acid (*r^2^* = 0.89, *p* = 0.02) (Figure 5b). On the contrary, MEhoneydew was significantly positively correlated with GABA (*r^2^* = 0.84, *p* = 0.04), AsA (*r^2^* = 0.94, *p* = 0.006), asparticacid (*r^2^* = 0.90, *p* = 0.01), serine (*r^2^* = 0.95, *p* = 0.004), and threonine (*r^2^* = 0.90, *p* = 0.01) (Figure 5b). MEorangered3 was significantly positively correlated with sucrose (*r^2^* = 0.88, *p* = 0.02), fructose (*r^2^* = 0.88, *p* = 0.02), glucose (*r^2^* = 0.89, *p* = 0.02), myo-inositol (*r^2^* = 0.81, *p* = 0.05), malic acid (*r^2^* = 0.94, *p* = 0.005), citric acid (*r^2^* = 0.83, *p* = 0.04), oxalic acid (*r^2^* = 0.86, *p* = 0.03), and arginine (*r^2^* = 0.94, *p* = 0.006), indicating that it is closely related to melon sweetness (Figure 5b). In addition, MEpink3 was significantly positively correlated with GABA (*r^2^* = 0.83, *p* = 0.04) and glutamic acid (*r^2^* = 0.92, *p* = 0.009) (Figure 5b). The gene networks were then constructed for these four typical modules (Figure 5c).

### Analysis of the key gene network and gene variations that affect primary metabolite abundance in H581 and HH09

**Figure 6:**
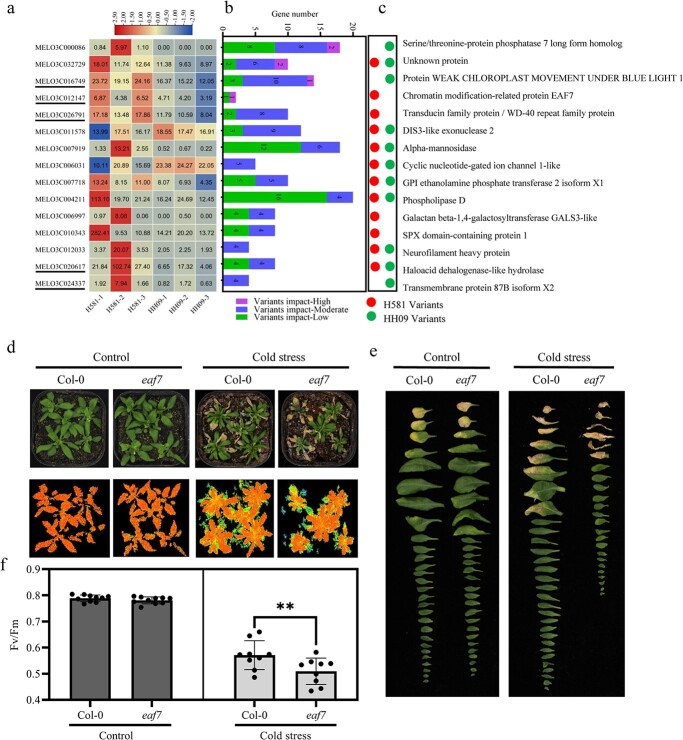
Sequence variation in candidate genes for cold tolerance and fruit quality (Primary metabolite) trade-offs. (a) The relative changes in expression levels of candidate genes with sequence variations. (b and c) Statistical analysis of sequence variations in genes in H581 and HH09. (d and e) Plant phenotypes and chlorophyll fluorescence (*Fv*/*Fm*) in wild-type *Arabidopsis thaliana* Col-0 and the *eaf7* mutant (SALK_020366C) grown under control and cold stress conditions, n = 9. Values are means ± SD, Student's *t* test. ^**^ indicates significant differences at *p* < 0.01. (f) Changes in plant growth in 4-week-old seedlings of Col-0 and the *eaf7* mutant under control and cold stress conditions.

To explore the key genes and regulatory networks that lead to differences in primary metabolite abundance between H581 and HH09, we analyzed the genotypic variations in key genes in four typical modules (Figure 5c). We found that 15 genes had more than four moderate-effect variants, of which four genes had high-effect variants in the two inbred lines (Figure 6b). Further comparison revealed that seven of these 15 genes had genotypic variations that were specific to H581 and HH09 (Figure 6c and Table S2). In addition, combining both gene expression (Figure 6a) and gene networks (Figure 5c), we identified *MELO3C016749.2*, *MELO3C012147.2*, *MELO3C026791.2*, *MELO3C010343.2*, and *MELO3C024337.2* as candidate genes in the trade-off between cold tolerance and fruit quality (primary metabolite) in melon. Among these genes, *MELO3C012147.2* (which we named *CmEAF7*), which encodes a subunit of the nucleosome acetyltransferase of H4 complex (NuA4), could be a candidate for the “ideal” trade-off gene between cold tolerance and fruit quality (Primary metabolite) in melon. In addition, we found that a four-base deletion occurred in the splice donor variant of the first intron of *CmEAF7* in H581, and this could affect splicing of the introns based on the resequencing results for H581 and HH09 (Figure 6c and Table S2).

**Figure 7:**
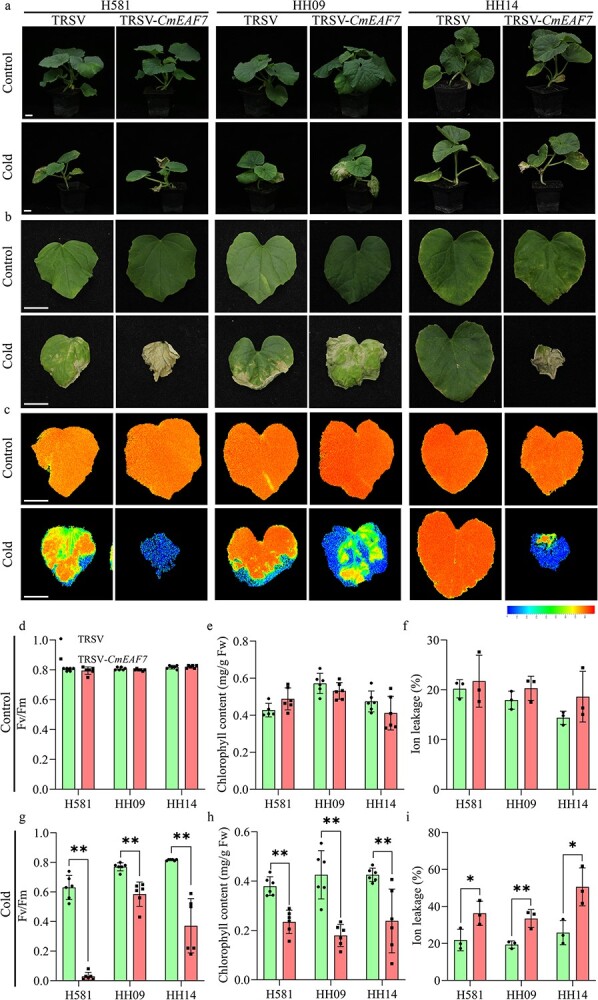
Cold tolerance in *CmEAF7-*silenced seedlings of melon lines H581, HH09, and HH14. (a) Phenotypes of *CmEAF7-*silenced plants. (b–c) Phenotypes and chlorophyll fluorescence in the first true leaves before and after cold treatment. (d and g) Statistical analysis of chlorophyll fluorescence (*Fv*/*Fm*) in the first true leaves before and after cold treatment, n = 6. (e and h) Statistical analysis of chlorophyll content in the first true leaves before and after cold treatment, n = 6. (f and i) Statistical analysis of ion leakage in the first true leaves before and after cold treatment (4°C for 16 h), n = 3. Seedlings of H581 and HH09 were treated as described in the materials and methods section. Values are means ± SD, Student's *t* test. ^*^ and ^**^ indicate significant differences at *p* < 0.05 and *p* < 0.01, respectively. Scale bars = 2 cm.

### *CmEAF7* improves both seedling cold tolerance and the contents of soluble sugars in melon fruits

To verify the functionality of *CmEAF7*, we used the *Arabidopsis* loss-of-function mutant *eaf7* which is caused by T-DNA insertion. The *AtEAF7* gene encodes SnRK2-substrate 1, and is the *Arabidopsis* homolog of *CmEAF7*. Under normal conditions, there were no significant differences in both growth and photosynthetic efficiency between *eaf7* and Col-0 plants (Figure 6D and E). We then performed cold treatment (−8°C for 6 h), followed by 4°C for 12 h in dark and the 22°C for 5 d under normal growth conditions, and the *eaf7* mutant plants showed sensitivity to low temperature (Figure 6D). The maximum photosynthetic efficiency (*Fv*/*Fw*) of the *eaf7* mutant was significantly lower than that of Col-0 plants (Figure 6E). We also evaluated plant growth by the number of leaves produced during the recovery period. After 5 days of recovery, the growth of *eaf7* plants was inhibited significantly, with fewer and smaller leaves compared with Col-0. There was no significant difference between *eaf7* and Col-0 plants grown without cold treatment. In summary, *AtEAF7* is a positive regulator of cold tolerance in *Arabidopsis*.

**Figure 8:**
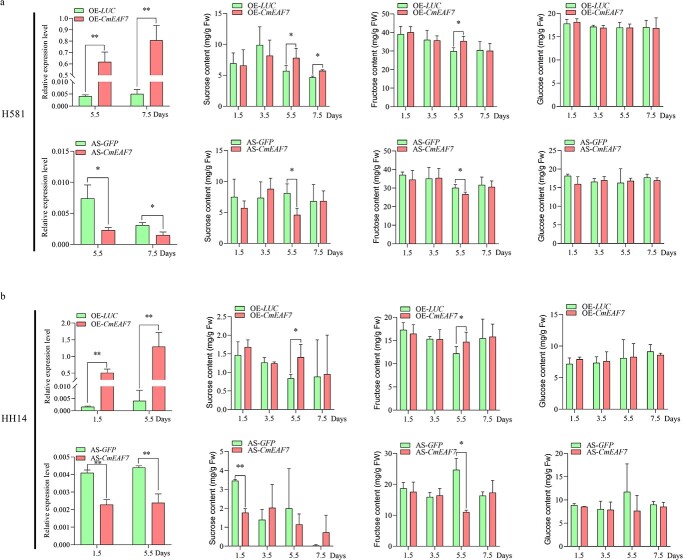
Transient expression of *CmEAF7* affects the accumulation of soluble sugars in melon fruits. (a) The contents of sucrose, fructose, and glucose after *CmEAF7* transient overexpression and silencing at 1.5, 3.5, 5.5, and 7.5 days after injection in H581 fruits, and expression of the *CmEAF7* gene at 5.5 and 7.5 days after injection. (b) The contents of sucrose, fructose, and glucose after *CmEAF7* transient overexpression and silencing at 1.5, 3.5, 5.5, and 7.5 days after injection in HH14 fruits, and expression of the *CmEAF7* gene at 1.5 and 5.5 d after injection. *CmActin* was used as a reference gene for normalization. OE-*LUC*: Transient overexpression of the empty vector; OE-*CmEAF7*: Transient overexpression of *CmEAF7*; AS*-GFP*: Transient silencing of the empty vector; AS-*CmEAF7*: Transient silencing of *CmEAF7*; n = 3–6; Values are means ± SD, Student's *t* test. ^*^ and ^**^ indicate significant differences at *p* < 0.05 and *p* < 0.01, respectively.

To further investigate whether *CmEAF7* plays a key role in cold tolerance and fruit quality in melon, we used VIGS (virus-induced gene silencing) technology to silence expression of *CmEAF7* in melon seedlings and transient overexpression and interference of *CmEAF7* by *A. tumefaciens* injection in melon fruits (Figure S3 and S4). After cold stress and recovery for 96 h, silencing of *CmEAF7* significantly decreased the cold tolerance of several melon genotypes, including H581, HH09, and HH14, with lower chlorophyll fluorescence (*Fv*/*Fm*), reduced chlorophyll content, and increased ion leakage (Figure 7 and S3). Additionally, the transient expression assay was performed on H581 and HH14 fruits, and the samples were collected at 1.5, 3.5, 5.5, and 7.5 days of after injection. The fluorescence (LUC and GFP) signals and *CmEAF7* gene expression levels were detected using an *in vivo* plant imaging system (Berthold LB985) and qRT-PCR, respectively. Our results showed that *CmEAF7* was successfully overexpressed (OE) or silenced (AS) in H581 and HH14 melon fruits (Figure 8a, 8b, and S4). Both the sucrose and fructose contents in OE-*CmEAF7* fruits significantly increased at 5.5 days after injection compared with OE-*LUC* fruits, while sucrose and fructose contents decreased significantly 5.5 days after injection in AS-*CmEAF7* fruits compared with AS-*GFP* fruits in H581 (Figure 8a). In addition, the sucrose content was higher at 7.5 days after injection in H581 fruits (Figure 8a), and the glucose content showed no difference in both the OE and AS fruits from H581 and HH14 (Figure 8a and 8b). Notably, similar results were obtained in another melon inbred line, HH14 (Figure 8b). These results indicate that *CmEAF7* can enhance both seedling cold tolerance and soluble sugar levels in melon fruits.

## Discussion

Melon (cultivars of *C. melo* var. *reticulatus*) is a commercially important plant that is grown for its fruits that are rich sources of carbohydrates and mineral nutrition. Melon is a cold-sensitive crop, and genetic improvement of melon cold tolerance requires elite genetic resources that enable the plants to tolerate cold temperatures without affecting fruit development and quality. The classic cold-responsive genes (e.g. *CBFs*) have been shown to promote cold tolerance in a variety of plant species in transgenic experiments, but they also have some negative effects such as inhibition of plant growth and organ development [25, 26]. Therefore, it is essential to identify genes that enhance cold tolerance without affecting plant growth and organ development. The trade-off between cold resistance and fruit quality (Primary metabolite) is a feature of the melon cultivars in the botanical group *C. melo* var. *reticulatus.* In this study, we used metabolomics, transcriptomics, and WGCNA to identify candidate trade-off regulatory genes that are involved in seedling cold tolerance and fruit quality (primary metabolite). Using transcriptome sequencing and genome resequencing of two melon genotypes with contrasting cold tolerance, we identified five genes (*MELO3C016749.2*, *MELO3C012147.2*, *MELO3C026791.2*, *MELO3C010343.2*, and *MELO3C024337.2*) as candidate genes that participate in the trade-off between cold stress and fruit quality (primary metabolite). Of these five genes, we identified sequence variants between the two genotypes in *MELO3C012147.2* (*CmEAF7*), and we showed that transient overexpression of this gene enhanced seedling cold tolerance and fruit quality (primary metabolite). *CmEAF7* could be an ideal trade-off gene for improving both cold tolerance and fruit quality (primary metabolite) in melon.

### The trade-off between cold tolerance and fruit quality (primary metabolite) in melon

Plant growth is always affected by various environmental stresses. Over time, plants have evolved to make a trade-off between survival and growth, such as between defense and growth [1, 2] and between stress and growth [3, 27, 28]. But does a trade-off exist between stress resistance and fruit quality? We investigated fruit quality in eight inbred melon lines that differ in their cold tolerance, and we found that fruit quality in the cold-sensitive melon varieties was generally higher than in the cold-resistant varieties (Figure 2a and b), which is consistent with the trade-off theory.

“Traditional” trade-off genes frequently have adverse effects on plant growth, development, quality, and/or yield while enhancing biotic and abiotic tolerance in plants [4, 7, 28, 29]. It is encouraging that more and more studies have identified some “ideal” trade-off genes that improve stress tolerance without affecting growth and development, and these could be ideal genes for use in plant breeding programs in the future [6, 8, 9, 30]. In this study, we found that there is a huge difference in fruit quality between the cold-resistant melon inbred lines H581 and HH09 (Figure 2a and b). Furthermore, transcriptomics, metabolomics, and genome resequencing were used to explore the genes involved in the trade-off between cold tolerance and fruit quality in the two melon lines. Five genes (*MELO3C016749.2*, *MELO3C012147.2*, *MELO3C026791.2*, *MELO3C010343.2*, and *MELO3C024337.2*) were predicted to be candidates for “ideal” trade-off genes (Figure 6), which could provide new genetic resources for the breeding of melon cultivars with high-quality fruits and enhanced tolerance to cold stress.

### CmEAF7 might balance cold-resistance and fruit quality (primary metabolite) by regulating chloroplast development and photosynthesis, as well as the ABA pathway, in melon

Posttranslational modification plays an important role in plant growth, development, and responses to environmental factors. Histone acetylation is an important posttranslational modification that participates in the regulation of gene transcriptional activity via chromatin remodeling [31]. Histone acetylation levels are antagonistically regulated by histone acetyltransferases and histone deacetylases. In plants, the nucleosome acetyltransferase of the H4 complex (NuA4), a member of the MYST family of histone acetyltransferases, participates in the regulation of various biological processes, as well as the hormone and stress response, by regulating the acetylation of histones H4, H2A, and H2A.Z [31, 32]. Interestingly, *MELO3C012147.2* (*CmEAF7*), which is a candidate for the “ideal” trade-off gene between cold tolerance and fruit quality in melon, encodes a subunit of the NuA4 complex. In addition, we found a four-base deletion in the splice donor variant of the first intron of *CmEAF7* in H581 that might affect splicing of the introns, based on the genomic resequencing results for H581 and HH09 (Figure 6c and Table S2).

Recent studies have shown that the NuA4 complex is required for plant chloroplast development and photosynthesis [32–34]. Similarly, our transcriptome data from H581 and HH09 shows a significant difference in chloroplast and photosynthesis genes (Figure 3a and b). In addition, *CmEAF7* was identified as a hub gene in the MEhoneydew module, and MEhoneydew was significantly positively correlated with GABA, AsA, serine, and threonine (Figure 5b and c). Therefore, we speculated that *CmEAF7* might balance cold tolerance and fruit quality in melon by regulating the acetylation of histone H4, and then in turn regulating the transcription of chloroplast and photosynthesis-related genes. Interestingly, we found two histone H4 genes (*MELO3C034813.2* and *MELO3C034814.2*) in the MEorangered3 module which were located at key nodes of the network, and the MEorangered3 module was significantly positively correlated with the major sugars (sucrose, fructose, and glucose) and organic acids (malic acid, citric acid, and oxalic acid) in melon fruit flesh (Figure 5b and c). Additional research is warranted to determine whether *CmEAF7* regulates sugar and organic acid metabolism through its involvement in the acetylation of these two H4 histones in melon.

Abscisic acid (ABA) is associated with fruit development and the stress response. *AtEAF7* (also known as *AtSNS1*), which is homologous to *CmEAF7*, inhibits ABA responses in *Arabidopsis* [31, 35]. Moreover, some key steps in ABA biosynthesis occur in chloroplasts [36]. Therefore, we compared the expression of the key genes in the ABA biosynthesis pathway in H581 and HH09 and found that these genes are differentially expressed in the two lines (Table S3 and Figure S2). To determine the biological function of *CmEAF7*, we performed transient expression experiments in melon seedlings and fruits, and the results showed that *CmEAF7* expression could improve seedling cold tolerance and fruit quality (Primary metabolite) in melon (Figures 7 and 8). However, whether *CmEAF7* balanced cold resistance and fruit quality (primary metabolite) in melon through the ABA pathway needs further experimental verification.

### Other candidate genes related to the trade-off between cold resistance and fruit quality (primary metabolite) in melon

It is well known that the source–sink relationship in plants is an important factor that affects the pattern of light energy capture and the distribution of photosynthates [37]. During fruit growth and development, the accumulation of organic nutrients in the fruit is based on the continuous input of carbohydrates from carbon fixation that occurs in the leaves [38]. Therefore, fruit quality and yield depend on leaf photosynthetic efficiency. Light-induced chloroplast movement is essential for efficient light utilization to improve photosynthetic efficiency in leaves [39, 40]. *WEB1*, a weak chloroplast movement under blue light 1 protein, can maintain the photorelocation speed of chloroplasts in *Arabidopsis* [41]. Moreover, *CmWEB1* (*MELO3C016749.2*) has DNA variation and a low level of expression in HH09 compared with H581 (Figure 6a), and it was also located in an important node in the MEhoneydew module (Figure 5c). These results suggest that *CmWEB1* is critical in regulating fruit quality in melon, and determining whether it is involved in the regulation of cold tolerance will require further study.

WD40 family genes are involved in plant development [42], abiotic stress [42, 43], the ABA response [43, 44], and metabolism [45]. HOS15, a WD40-repeat protein, plays a key role in gene activation/repression via histone acetylation or deacetylation in plants and in improving low temperature tolerance in *Arabidopsis* [46]. Also, SlWD40 (WD-40 repeats), a fruit ripening regulator in tomato, also affects tomato fruit quality [47]. In addition, the MYB/bHLH/WD40 regulatory complex affects fruit quality in strawberry and blueberry by controlling proanthocyanidin biosynthesis [48, 49]. Our research identified the WD-40 repeat family protein gene *MELO3C026791.2* (*CmWD40*) as a hub gene within the MEhoneydew module (Figure 5c), and it had a moderate variant in H581 and showed differential expression between H581 and HH09 (Figure 6a-c). We therefore speculated that *CmWD40* could also regulate the trade-off between seedling cold-resistance and fruit quality in melon.

Phosphate availability is a major factor that limits plant growth, development, and productivity [50, 51]. Previous studies have shown that *AtSPX1* and *OsSPX1*, homologs of *CmSPX1*, are involved in regulating the plant response to phosphorus starvation in *Arabidopsis* [52, 53] and rice [53, 54], respectively. In addition, *OsSPX1* positively regulates seedling cold tolerance in rice [55], *Arabidopsis*, and tobacco [56]. In this study, we found that there are sequence variants in *CmSPX1* (*MELO3C010343.2*) between H581 and HH09, and that this gene is located in key nodes of the interaction network in MEpink3 (Figures 5c and 6a–c). Therefore, *CmSPX1* is considered to be a candidate gene involved in regulating seedling cold tolerance and fruit quality in melon. However, the function of *MELO3C024337.2*, which shows sequence variation between HH09 and H581, in MEorangered3 is unclear.

## Conclusion

In this study, a total of 31 primary metabolites that include soluble sugars, organic acids, and amino acids were detected in the fruits of eight melon lines that differ with respect to seedling cold tolerance. The primary metabolomic and transcriptomic data for the melon lines H581 and HH09 were subjected to WGCNA, and five key genes with specific genotypic sequence variations between H581 and HH09 were identified as candidate genes that could be involved in regulating fruit quality (Primary metabolite) in melon. We used VIGS technology to silence *CmEAF7* expression in melon seedlings and transient overexpression and interference of *CmEAF7* by *A. tumefaciens* infiltration in melon fruits. Taken together, the results of our study lead to the identification of a trade-off gene, *CmEAF7*, that promotes both seedling cold tolerance and fruit quality (Primary metabolite)in melon.

## Materials and methods

### Plant materials

Eight inbred melon lines (H581, H1037, H929, HH14, HH09, HH36, HH94, and HH90) were used in this study. All of them are classified as *C. melo* var. *reticulatus* based on the classification scheme suggested by Pitrat [57]; the related information is given in Table S1. Seeds of the eight melon genotypes were sown in multiplug trays in a growth chamber with a photoperiod of 16 h/8 h (light/dark) and a temperature range of 28/18°C (day/night). Some seedlings at the two-leaf stage were planted in a greenhouse for fruit development, and the fruits were harvested at about 40 days after pollination. Samples of fresh fruit flesh were collected, flash frozen in liquid nitrogen, and stored at −80°C prior to use in transcriptomic and metabolomic analyses. Fruit flesh of the cold-resistant line H581 and the moderately cold-resistant line HH09 were used to construct RNA-seq libraries, with three biological replicates. There were four biological replicates for GC–MS analysis, and in both cases, each biological replicate consisted of three fruits from different plants.

### Seedling cold treatment and identification of cold resistance

Two-leaf seedlings were used in the cold treatment experiments. Seedlings with developmental uniformity were treated in a climatic chamber at 4°C for 48 h, followed by recovery at normal conditions for 72 h. The chilling injury class was then determined by scoring the phenotype of the first true leaf, and the chilling injury index (CII) was calculated according to Hou et al. [18], although the number of injury classes was increased from five to nine. Three biological replicates were performed for CII and each replicate contained 15 individuals. At the same time, chlorophyll fluorescence (*Fv*/*Fm*) was examined on the first true leaf of five cold-treated plants from the eight melon genotypes using a FluorCAM 800MF (Photon Systems Instruments, Brno, Czech Republic), with the untreated plants as the controls.

The wild-type *Arabidopsis thaliana* ecotype Col-0 was used in this study. The *Arabidopsis* mutant *eaf7* (SALK_020366C) (*AT1G26470*, which encodes SnRK2-substrate 1, is the homologous gene of *CmEAF7*) was purchased from the AraShare Center (https://www.arashare.cn/index). The wild-type Col-0 and *eaf7* mutant seedlings were grown in a plant growth chamber at 22°C under a 16-h day/8-h night photoperiod with an illumination of 22 000 lux at 75% relative humidity. For cold stress treatment, 3-week-old WT and mutant plants grown in soil were treated at –8°C for 6 h at an illumination of 10 000 lux, and then allowed to recover for 12 h in darkness at 4°C. After 5 days of growth under normal conditions, phenotypic and chlorophyll fluorescence (*Fv*/*Fm*) data were collected.

### Metabolite analysis of melon flesh by GC–MS

Metabolite analysis was performed using gas chromatography–mass spectrometry (GC–MS) on melon flesh. Samples from the eight melon genotypes were processed and extracted as described by Lisec et al. [58]. Samples were ground to a powder in liquid nitrogen, and 200 mg of the powder was extracted with 1.5 ml of methanol. Then, 63 μl of the internal standard (0.2 mg ribitol ml^−1^ in MilliQ water) was added for quantification. A total of 48 standards were performed for targeted metabolomics in four biological replicates, with each biological replicate consisting of three fruits from different plants. Sample derivatization, GC–MS, and compound identification were performed using the protocols established by Lisec et al. [58]. Heatmaps and clustering of metabolic data were performed using TBtools 1.33210.0.0 [59], and Pearson’s correlation analysis was performed using R4.2.0 software (https://www.r-project.org/). Principal component analysis (PCA) was performed using Past 2.17 (PAleontological STatistics) software, which is a paleontological statistics software package for education and data analysis.

### Transcriptome sequencing of melon flesh

Fruit flesh of the cold resistant line H581 and the moderately cold resistant line HH09 were used to construct RNA-seq libraries. There were three biological replicates, and each biological replicate consisted of three fruits from different plants. RNA was extracted using TRIzol reagent (Invitrogen). The stranded mRNA libraries were sequenced on Illumina HiSeq™ 2500/MiSeq platforms in 150-bp paired-end read mode. The raw data quality were assessed using FASTQC v.0.11.8 software [60] and trimming of Illumina adapters and bases with quality scores <20 was performed with Cutadapt [61]. Next, the clean reads were mapped to the melon reference genome (DHL92 genome 3.6.1) using Hisat2 v2.1.0 [62]. Additionally, the differentially expressed genes (DEGs) were further analyzed using the Gene Ontology (GO) and Kyoto Encyclopedia of Genes and Genomes (KEGG) databases to assess their functional enrichment using the phyper function in R software. The raw RNA-seq data generated in this study is available at the NCBI-SRA database (https://www.ncbi.nlm.nih.gov/sra) under accession number PRJNA911592.

### Construction of correlated gene networks by weighted correlation network analysis

Weighted correlation network analysis was performed with the weighted correlation network analysis (WGCNA) R4.2.0 package [63]. An adjacency matrix was constructed using normalized FPKM (fragments per kilobase of exon per million mapped fragments) values for genes in the transcriptome. The contents of 14 metabolites were used as phenotypic data, and the association analysis between phenotypes and gene modules was performed with the WGCNA package using the default parameters. The adjacency matrix was then converted into a topological overlap matrix. In the coexpression networks, the genes in the same module have identical expression patterns.

### Analysis of genetic variants in two typical melon lines

The raw resequencing data from the two inbred melon lines H581 and HH09 has been deposited in the NCBI-SRA database (https://www.ncbi.nlm.nih.gov/sra) under accession numbers PRJNA911606 (H581) and PRJNA808180 (HH09). Based on the melon reference genome (DHL92 v3.6.1), we performed variation analysis between H581 and HH09 using GATK v4.0 [64]. The effects of the genetic variants were annotated and predicted by SnpEff v5.1 [65].

### Virus-induced gene silencing (VIGS) in melon seedlings

The VIGS experiment was performed according to the method of Dong et al. [66]. The pTRSV1 and pTRSV2 vectors were used for VIGS. A 269-bp fragment of *CmEAF7* was amplified from HH09 cDNA by PCR using the *CmEAF7*-TRSV2-F and *CmEAF7*-TRSV2-R primer pair, cloned onto the pTRSV2 vector, and transformed into *Agrobacterium tumefaciens* strain GV3101. The resulting strain was named TRSV-*CmEAF7* (Table S4). Seeds of H581, HH09, and HH14 were germinated on half-strength MS medium, and vacuum infiltration was carried out when the roots reached 1 to 1.5 cm in length. Following 4 days of cocultivation at 25°C in the dark, the seedlings were transferred to soil. When the seedlings had grown three true leaves, seedlings of H581 and HH09 were treated at 4°C for 60 h, then 25°C for 6 h, followed by 0°C for 48 h and 25°C for 96 h, and the seedlings of HH14 were treated at 4°C for 60 h, followed by 25°C for 96 h.

### Transient transformation of melon fruits

The full coding sequence (CDS) of *CmEAF7* without the stop codon was cloned in the sense orientation into the plant overexpression vector pCAMBIA3301 containing the LUC (luciferase) tag. The resulting vector was named OE-*CmEAF7*. Similarly, the *CmEAF7* CDS was also cloned into the pRI101 vector with a GFP tag as the antisense construct and named AS-*CmEAF7*. Both vectors were transformed into *A. tumefaciens* strain EHA105. H581 and HH14 fruits (20 to 25 days after pollination) were used in the transient expression experiments following the method of Duan et al. [67]. Samples were harvested at 1.5, 3.5, 5.5, and 7.5 days after injection. The fruit flesh around the injection site was excised with a 1 cm diameter punch, and the LUC and GFP fluorescence signals were analyzed. There were three to six biological replicates for each treatment.

### Measurement of physiological and biochemical indexes in melon plants

Ion leakage assays were performed as described by Jiang et al. [68]. First, six leaf discs from the first true leaf were collected into 15 ml tubes containing 8 ml deionized water, shaken for 15 min (200 r/min) at room temperature, and the electrical conductivity was measured as S1. Second, the tubes containing the samples were boiled for 20 min and then shaken for 20 min at room temperature. The electrical conductivity of the solution was measured as S2. Deionized water was used as a blank control and denoted as S0. The ion leakage was calculated using the formula (S1-S0)/(S2-S0).

After the plants had recovered from the cold treatment, the chlorophyll contents were determined. Eight leaf discs (about 0.1 g) from the first true leaf were collected into 2-ml tubes, and 1.8 ml extraction solution (anhydrous ethanol/acetone/deionized water = 4.5:4.5:1) was added to each tube. The samples were extracted at 4°C in dark for 24 h. The absorbances at 645 nm and 663 nm were measured, and the total chlorophyll content was calculated using the following formula: (20.29 × A_645_ + 8.05 × A_663_) × 1.8/ (0.1 × 1000).

The LUC and GFP fluorescence signal intensities of the samples were measured using an *in vivo* fluorescence imager (Berthold LB985; Germany). Glucose, fructose, and sucrose contents of the melon flesh samples were determined using the D-Glucose Content Assay Kit (Cat. AKSU001M; boxbio; Beijing, China), the Plant Fructose Content Assay Kit (Cat. AKPL007M; boxbio; China), and the Plant Sucrose Content Assay Kit (Cat. AKPL006M; boxbio; China), respectively.

### Quantitative real-time PCR analysis

Total RNA extraction and gene expression analysis were performed as in our previous study [27]. The names and nucleotide sequences of the qRT-PCR primers are given in Table S4. *CmActin* was used as a reference gene for normalization. qRT-PCR assays were performed using a Bio-Rad Thermal Cycler, and the relative gene expression was calculated using the 2^-ΔCt^ method.

### Statistical analysis

All data represent the means ± SD of three or more biological replicates. The significant differences were analyzed using Student's *t* tests or one-way analysis of variance (ANOVA). ^*^ and ^**^ indicates significant differences at *p* < 0.05 and *p* < 0.01, respectively, and the same letter above a column indicates there was no significant difference at *p* < 0.05 as determined by ANOVA.

## Acknowledgements

This work was supported by the National Natural Science Foundation of China (Grant numbers 31872101 and 32072564), the Henan Special Funds for Major Science and Technology (221100110400), the Excellent Youth Foundation of Henan Scientific Committee (222300420009), and the Foundation for Young Talents of Henan Agricultural University (30500728). We thank Dr. Meng Li for kindly providing the transient transformation vector.

## Author contributions

Lili Li: Conceptualization, Methodology, Validation, Investigation, Writing - Original Draft, Visualization. Qiong Li: Methodology, Software, Investigation, Visualization. Bin Chen: Methodology, Software, Investigation, Visualization. Jiyu Wang: Validation, Investigation. Fei Ding: Software, Investigation, Visualization, Data Curation. Panqiao Wang: Software, Data Curation, Visualization. Xiuyue Zhang: Investigation, Visualization. Juan Hou: Investigation, Data Curation. Renren Luo: Investigation, Data Curation. Xiang Li: Investigation, Data Curation. Jingwen Zheng: Software, Data Curation. Sen Yang: Data Curation, Review & Editing. Luming Yang: Resources, Review & Editing. Lei Zhu: Resources, Review & Editing. Shouru Sun: Resources, Review & Editing. Changsheng Ma: Resources, Review & Editing. Qin Li: Resources, Review & Editing. Ying Li: Conceptualization, Methodology, Validation, Formal analysis, Writing - Review & Editing, Supervision, Project administration, Funding acquisition. Jianbin Hu: Conceptualization, Resources, Writing - Review & Editing, Supervision, Project administration, Funding acquisition.

## Data availability

All data are included in the paper and in the Supplementary Materials published online.

## Conflicts of interest statement

The authors declare that they have no known competing financial interests or personal relationships that could have appeared to influence the outcome of the research reported in this paper.

## Supplementary Data

Supplementary data is available at *Horticulture Research* online.

## Supplementary Material

Web_Material_uhad093

## References

[1] Huot B , Yao J, Montgomery BL et al. Growth-defense tradeoffs in plants: a balancing act to optimize fitness. Mol Plant. 2014;7:1267–87. 24777989 10.1093/mp/ssu049 PMC4168297

[2] Zust T , Agrawal AA. Trade-offs between plant growth and defense against insect herbivory: an emerging mechanistic synthesis. Annu Rev Plant Biol. 2017;68:513–34. 28142282 10.1146/annurev-arplant-042916-040856

[3] Ding YL , Yang SH. Surviving and thriving: how plants perceive and respond to temperature stress. Dev Cell. 2022;57:947–58. 35417676 10.1016/j.devcel.2022.03.010

[4] Goto S , Sasakura-Shimoda F, Suetsugu M et al. Development of disease-resistant rice by optimized expression of WRKY45. Plant Biotechnol J. 2015;13:753–65. 25487714 10.1111/pbi.12303

[5] Cerrudo I , Caliri-Ortiz ME, Keller MM et al. Exploring growth-defence trade-offs in *Arabidopsis*: phytochrome B inactivation requires JAZ10 to suppress plant immunity but not to trigger shade-avoidance responses. Plant Cell Environ. 2017;40:635–44. 27943325 10.1111/pce.12877

[6] Deng Y , Zhai K, Xie Z et al. Epigenetic regulation of antagonistic receptors confers rice blast resistance with yield balance. Science. 2017;355:962–5. 28154240 10.1126/science.aai8898

[7] Ke Y , Yuan M, Liu H et al. The versatile functions of OsALDH2B1 provide a genic basis for growth-defense trade-offs in rice. Proc Natl Acad Sci U S A. 2020;117:3867–73. 32024752 10.1073/pnas.1918994117 PMC7035479

[8] Liu MM , Shi ZY, Zhang XH et al. Inducible overexpression of ideal plant Architecture1 improves both yield and disease resistance in rice. Nature Plants. 2019;5:389–400. 30886331 10.1038/s41477-019-0383-2

[9] Wang H , Li Y, Chern M et al. Suppression of rice miR168 improves yield, flowering time and immunity. Nature Plants. 2021;7:129–36. 33594262 10.1038/s41477-021-00852-x

[10] Pearce RS . Plant freezing and damage. Ann Bot. 2001;87:417–24.

[11] Clj A , Gcd A, Vkwa B. Cold acclimation and prospects for cold-resilient crops. Plant Stress. 2021;2:100028.

[12] Zhao GW , Lian Q, Zhang ZH et al. A comprehensive genome variation map of melon identifies multiple domestication events and loci influencing agronomic traits. Nat Genet. 2019;51:1607–15. 31676864 10.1038/s41588-019-0522-8

[13] Solmaz I , Kacar YA, Simsek O et al. Genetic characterization of Turkish Snake melon (*Cucumis melo* L. subsp *Melo flexuosus* group) accessions revealed by SSR markers. Biochem Genet. 2016;54:534–43. 27193591 10.1007/s10528-016-9739-8

[14] Korkmaz A , Dufault RJ. Developmental consequences of cold temperature stress at transplanting on seedling and field growth and yield. II. Melon. J Am Soc Hortic Sci. 2001;126:410–3.

[15] Korkmaz A , Dufault RJ. Influence of short-term cyclic cold temperature stress on melon and honeydew yield. HortTechnology. 2003;13:67–70.

[16] Li M , Duan XY, Wang Q et al. A new morphological method to identify cold tolerance of melon at seedling stage. Funct Plant Biol. 2020;47:80–90. 10.1071/FP19163 31813411

[17] Ding YL , Shi YT, Yang SH. Advances and challenges in uncovering cold tolerance regulatory mechanisms in plants. New Phytol. 2019;222:1690–704. 30664232 10.1111/nph.15696

[18] Hou J , Zhou YF, Gao LY et al. Dissecting the genetic architecture of melon chilling tolerance at the seedling stage by association mapping and identification of the elite alleles. Front Plant Sci. 2018;9:1577. 30429864 10.3389/fpls.2018.01577 PMC6220089

[19] Song ZC , Wang WP, Shi LX et al. Identification of QTLs controlling low-temperature tolerance during the germination stage in cucumber (*Cucumis sativus* L.). Plant Breed. 2018;137:629–37.

[20] Yagcioglu M , Jiang B, Wang P et al. QTL mapping of low temperature germination ability in cucumber. Euphytica. 2019;215:84.

[21] Xu Y , Guo SR, Shu S et al. Construction of a genetic linkage map of rootstock-used pumpkin using SSR markers and QTL analysis for cold tolerance. Sci Hortic. 2017;220:107–13.

[22] Li M , Duan XY, Gao G et al. CmABF1 and CmCBF4 cooperatively regulate putrescine synthesis to improve cold tolerance of melon seedlings. Hortic Res. 2022;9:uhac002.35147169

[23] Wei SB , Li X, Lu ZF et al. A transcriptional regulator that boosts grain yields and shortens the growth duration of rice. Science. 2022;377:eabi8455. 35862527 10.1126/science.abi8455

[24] Sun XP , Xiang YL, Dou NN et al. The role of transposon inverted repeats in balancing drought tolerance and yield-related traits in maize. Nat Biotechnol. 2022;41:120–7. 36229611 10.1038/s41587-022-01470-4

[25] Jaglo-Ottosen KR , Gilmour SJ, Zarka DG et al. *Arabidopsis* CBF1 overexpression induces COR genes and enhances freezing tolerance. Science. 1998;280:104–6. 9525853 10.1126/science.280.5360.104

[26] Gilmour SJ , Sebolt AM, Salazar MP et al. Overexpression of the *Arabidopsis* CBF3 transcriptional activator mimics multiple biochemical changes associated with cold acclimation. Plant Physiol. 2000;124:1854–65. 11115899 10.1104/pp.124.4.1854 PMC59880

[27] Ding F , Qiang X, Jia ZQ et al. Knockout of a novel salt responsive gene SlABIG1 enhance salinity tolerance in tomato. Environ Exp Bot. 2022;200:104903.

[28] Zhang H , Zhao Y, Zhu JK. Thriving under stress: how plants balance growth and the stress response. Dev Cell. 2020;55:529–43. 33290694 10.1016/j.devcel.2020.10.012

[29] Liu WC , Song RF, Zheng SQ et al. Coordination of plant growth and abiotic stress responses by tryptophan synthase beta subunit 1 through modulation of tryptophan and ABA homeostasis in *Arabidopsis*. Mol Plant. 2022;15:973–90. 35488429 10.1016/j.molp.2022.04.009

[30] Wang J , Zhou L, Shi H et al. A single transcription factor promotes both yield and immunity in rice. Science. 2018;361:1026–8. 30190406 10.1126/science.aat7675

[31] Espinosa-Cores L , Bouza-Morcillo L, Barrero-Gil J et al. Insights into the function of the NuA4 complex in plants. Front Plant Sci. 2020;11:125. 32153620 10.3389/fpls.2020.00125 PMC7047200

[32] Bieluszewski T , Sura W, Dziegielewski W et al. NuA4 and H2A.Z control environmental responses and autotrophic growth in *Arabidopsis*. Nature Communications. 2022;13:277. 10.1038/s41467-021-27882-5 PMC8755797 35022409

[33] Ding B , Xie H, Zhang K et al. Nuclear EPL-HAM complex is essential for the development of chloroplasts. J Gene Genom. 2022;49:1165–8. 10.1016/j.jgg.2022.04.006 35489697

[34] Zhou JX , Su XM, Zheng SY et al. The *Arabidopsis* NuA4 histone acetyltransferase complex is required for chlorophyll biosynthesis and photosynthesis. J Integr Plant Biol. 2022;64:901–14. 35043580 10.1111/jipb.13227

[35] Umezawa T , Sugiyama N, Takahashi F et al. Genetics and phosphoproteomics reveal a protein phosphorylation network in the abscisic acid signaling pathway in *Arabidopsis thaliana*. Sci Signal. 2013;6:rs8. 23572148 10.1126/scisignal.2003509

[36] Gong ZZ , Xiong LM, Shi HZ et al. Plant abiotic stress response and nutrient use efficiency. Science China-Life Sciences. 2020;63:635–74. 32246404 10.1007/s11427-020-1683-x

[37] Smith MR , Rao IM, Merchant A. Source-sink relationships in crop plants and their influence on yield development and nutritional quality. Front Plant Sci. 2018;9:1889. 30619435 10.3389/fpls.2018.01889 PMC6306447

[38] Hernandez-Santana V , Perez-Arcoiza A, Gomez-Jimenez MC et al. Disentangling the link between leaf photosynthesis and turgor in fruit growth. Plant J. 2021;107:1788–801. 34250661 10.1111/tpj.15418

[39] Suetsugu N , Wada M. Chloroplast photorelocation movement: a sophisticated strategy for chloroplasts to perform efficient photosynthesis. Advances in Photosynthesis—Fundamental Aspects. 2012;215-234.

[40] Kihara M , Ushijima T, Yamagata Y et al. Light-induced chloroplast movements in Oryza species. J Plant Res. 2020;133:525–35. 32303870 10.1007/s10265-020-01189-w

[41] Kodama Y , Suetsugu N, Kong SG et al. Two interacting coiled-coil proteins, WEB1 and PMI2, maintain the chloroplast photorelocation movement velocity in Arabidopsis. Proc Natl Acad Sci U S A. 2010;107:19591–6. 20974974 10.1073/pnas.1007836107 PMC2984159

[42] Tan L , Salih H, Htet NNW et al. Genomic analysis of WD40 protein family in the mango reveals a TTG1 protein enhances root growth and abiotic tolerance in Arabidopsis. Sci Rep. 2021;11:2266. 33500544 10.1038/s41598-021-81969-z PMC7838414

[43] Li Q , Zhao P, Li J et al. Genome-wide analysis of the WD-repeat protein family in cucumber and Arabidopsis. Mol Gen Genomics. 2014;289:103–24. 10.1007/s00438-013-0789-x 24292651

[44] Xu XZ , Wan W, Jiang GB et al. Nucleocytoplasmic trafficking of the *Arabidopsis* WD40 repeat protein XIW1 regulates ABI5 stability and abscisic acid responses. Mol Plant. 2019;12:1598–611. 31295628 10.1016/j.molp.2019.07.001

[45] Yang XH , Wang JR, Xia XZ et al. OsTTG1, a WD40 repeat gene, regulates anthocyanin biosynthesis in rice. Plant J. 2021;107:198–214. 33884679 10.1111/tpj.15285

[46] Zhu J , Jeong JC, Zhu Y et al. Involvement of *Arabidopsis* HOS15 in histone deacetylation and cold tolerance. Proc Natl Acad Sci U S A. 2008;105:4945–50. 18356294 10.1073/pnas.0801029105 PMC2290775

[47] Zhu F , Jadhav SS, Tohge T et al. A comparative transcriptomics and eQTL approach identifies SlWD40 as a tomato fruit ripening regulator. Plant Physiol. 2022;190:250–66. 35512210 10.1093/plphys/kiac200 PMC9434188

[48] Schaart JG , Dubos C, Romero De La Fuente I et al. Identification and characterization of MYB-bHLH-WD40 regulatory complexes controlling proanthocyanidin biosynthesis in strawberry (Fragaria x ananassa) fruits. New Phytol. 2013;197:454–67. 23157553 10.1111/nph.12017

[49] Zhao M , Li J, Zhu L et al. Identification and characterization of MYB-bHLH-WD40 regulatory complex members controlling anthocyanidin biosynthesis in blueberry fruits development. Genes (Basel). 2019;10. 10.3390/genes10070496 PMC6678982 31261791

[50] Rouached H , Arpat AB, Poirier Y. Regulation of phosphate starvation responses in plants: signaling players and cross-talks. Mol Plant. 2010;3:288–99. 20142416 10.1093/mp/ssp120

[51] Zhang K , Song Q, Wei Q et al. Down-regulation of OsSPX1 caused semi-male sterility, resulting in reduction of grain yield in rice. Plant Biotechnol J. 2016;14:1661–72. 26806409 10.1111/pbi.12527 PMC5066639

[52] Puga MI , Mateos I, Charukesi R et al. SPX1 is a phosphate-dependent inhibitor of PHOSPHATE STARVATION RESPONSE 1 in Arabidopsis. Proc Natl Acad Sci U S A. 2014;111:14947–52. 25271326 10.1073/pnas.1404654111 PMC4205628

[53] Liu N , Shang WY, Li C et al. Evolution of the SPX gene family in plants and its role in the response mechanism to phosphorus stress. Open Biol. 2018;8:170231. 29298909 10.1098/rsob.170231 PMC5795055

[54] Wang C , Ying S, Huang HJ et al. Involvement of OsSPX1 in phosphate homeostasis in rice. Plant J. 2009;57:895–904. 19000161 10.1111/j.1365-313X.2008.03734.x

[55] Wang C , Wei Q, Zhang K et al. Down-regulation of OsSPX1 causes high sensitivity to cold and oxidative stresses in rice seedlings. PLoS One. 2013;8:e81849. 24312593 10.1371/journal.pone.0081849 PMC3849359

[56] Zhao L , Liu FX, Xu WY et al. Increased expression of OsSPX1 enhances cold/subfreezing tolerance in tobacco and *Arabidopsis thaliana*. Plant Biotechnol J. 2009;7:550–61. 19508276 10.1111/j.1467-7652.2009.00423.x

[57] Pitrat M . Melon. In: Vegetables I. Springer, 2008,283–315.

[58] Lisec J , Schauer N, Kopka J et al. Gas chromatography mass spectrometry-based metabolite profiling in plants. Nat Protoc. 2006;1:387–96. 17406261 10.1038/nprot.2006.59

[59] Chen CJ , Chen H, Zhang Y et al. TBtools: an integrative toolkit developed for interactive analyses of big biological data. Mol Plant. 2020;13:1194–202. 32585190 10.1016/j.molp.2020.06.009

[60] Andrews S . FastQC: A Quality Control Tool for High Throughput Sequence Data. Babraham Institute, Cambridge, United Kingdom: Babraham Bioinformatics; 2010.

[61] Martin M . Cutadapt removes adapter sequences from high-throughput sequencing reads. EMBnet J. 2011;17:10–2.

[62] Kim D , Landmead B, Salzberg SL. HISAT: a fast spliced aligner with low memory requirements. Nat Methods. 2015;12:357–60. 25751142 10.1038/nmeth.3317 PMC4655817

[63] Langfelder P , Horvath S. WGCNA: an R package for weighted correlation network analysis. Bmc Bioinformatics. 2008;9:559. 19114008 10.1186/1471-2105-9-559 PMC2631488

[64] McCormick RF , Truong SK, Mullet JE. RIG: recalibration and interrelation of genomic sequence data with the GATK. G3 (Bethesda). 2015;5:655–65. 25681258 10.1534/g3.115.017012 PMC4390580

[65] Cingolani P , Platts A, Wang LL et al. A program for annotating and predicting the effects of single nucleotide polymorphisms, SnpEff: SNPs in the genome of *Drosophila melanogaster* strain w(1118); iso-2; iso-3. Fly. 2012;6:80–92. 22728672 10.4161/fly.19695 PMC3679285

[66] Dong MM , Xue SD, Bartholomew ES et al. Transcriptomic and functional analysis provides molecular insights into multicellular trichome development. Plant Physiol. 2022;189:301–14. 35171294 10.1093/plphys/kiac050 PMC9070826

[67] Duan XY , Cai J, Zhao YP et al. Transcriptome and metabolomics analysis revealed that CmWRKY49 regulating CmPSY1 promotes β-carotene accumulation in orange fleshed oriental melon. Hortic Plant J. 2022;8:650–66.

[68] Jiang BC , Shi YT, Zhang XY et al. PIF3 is a negative regulator of the CBF pathway and freezing tolerance in *Arabidopsis*. Proc Natl Acad Sci U S A. 2017;114:E6695–702. 28739888 10.1073/pnas.1706226114 PMC5559041

