# Supplementary material for: Identification of candidate genes that regulate the trade-off between seedling cold tolerance and fruit quality in melon (Cucumis melo L.)
Source: Hortic Res. 2023 May 9;10(7):uhad093. doi: 10.1093/hr/uhad093 (PMC10321389; doi:10.1093/hr/uhad093)
Supplement: Web_Material_uhad093 [file web_material_uhad093.zip › Supplementary Figures.docx]

**Supplementary Figures**


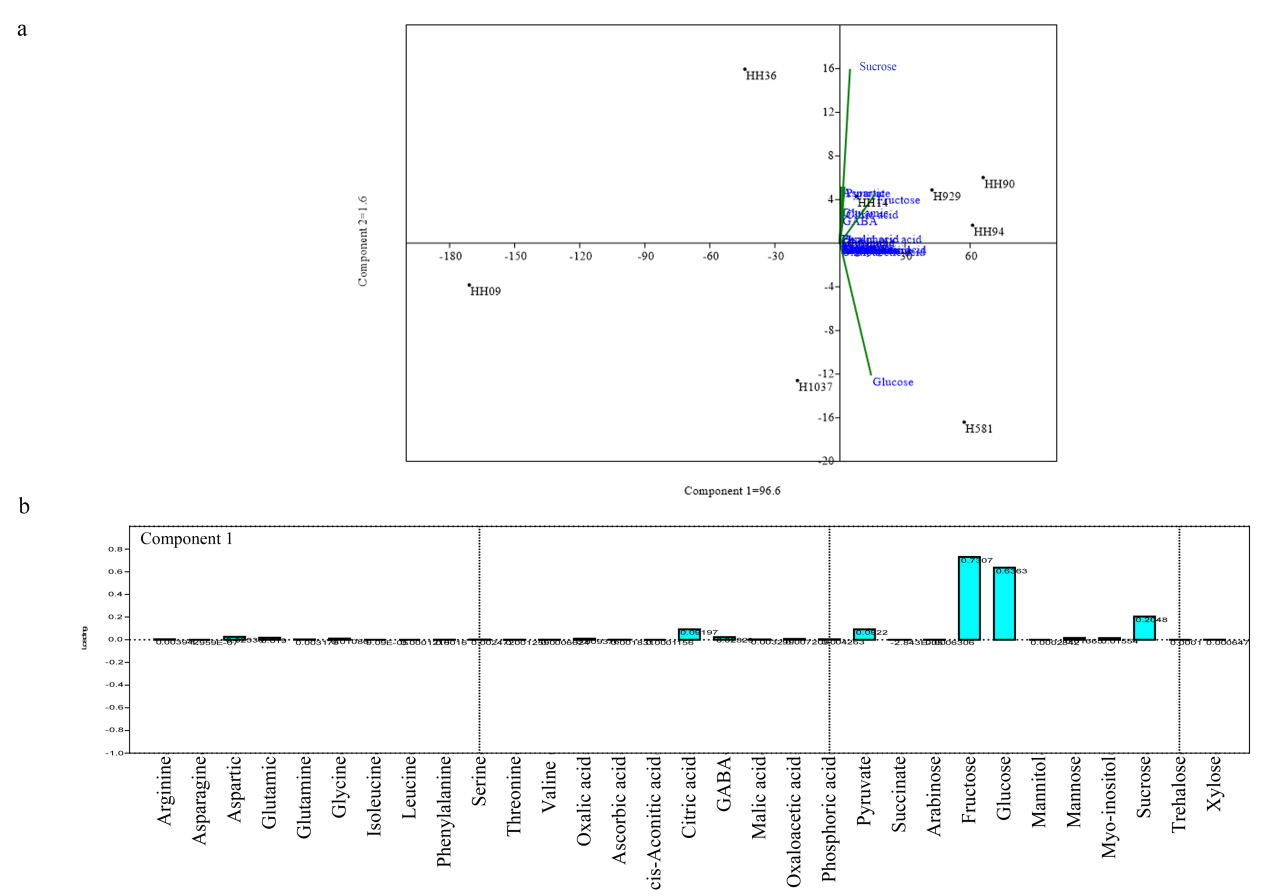


Figure S1

Figure S1. Principal component analysis (PCA) of GC-MS data from eight inbred melon lines.(a) PCA of eight inbred melon lines. (b) The loadings of PCA1 from the fruit fresh of the eight inbred lines.


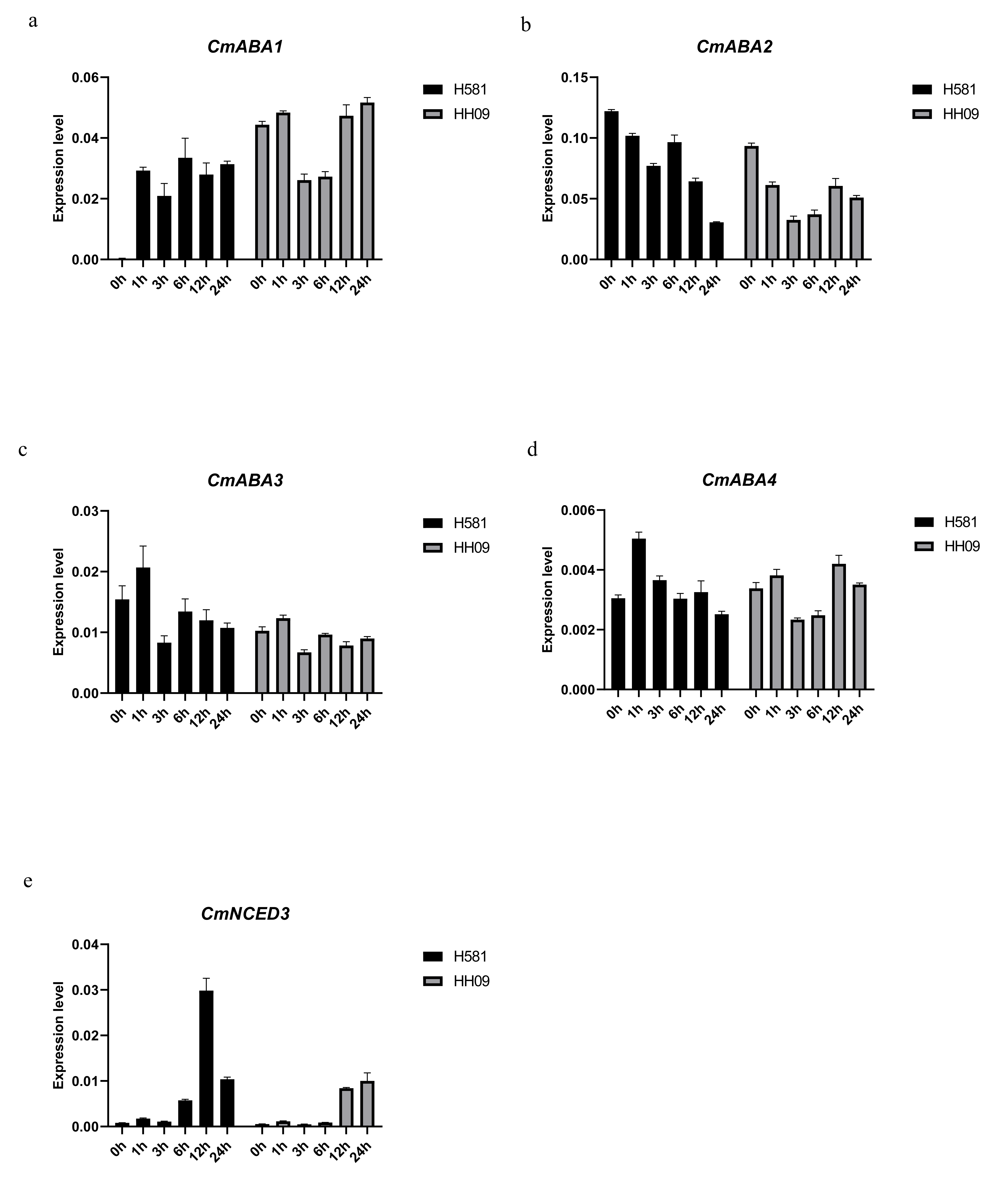


Figure S2

Figure S2. The expression levels of key ABA biosynthesis pathway genes were verified by qRT-PCR.


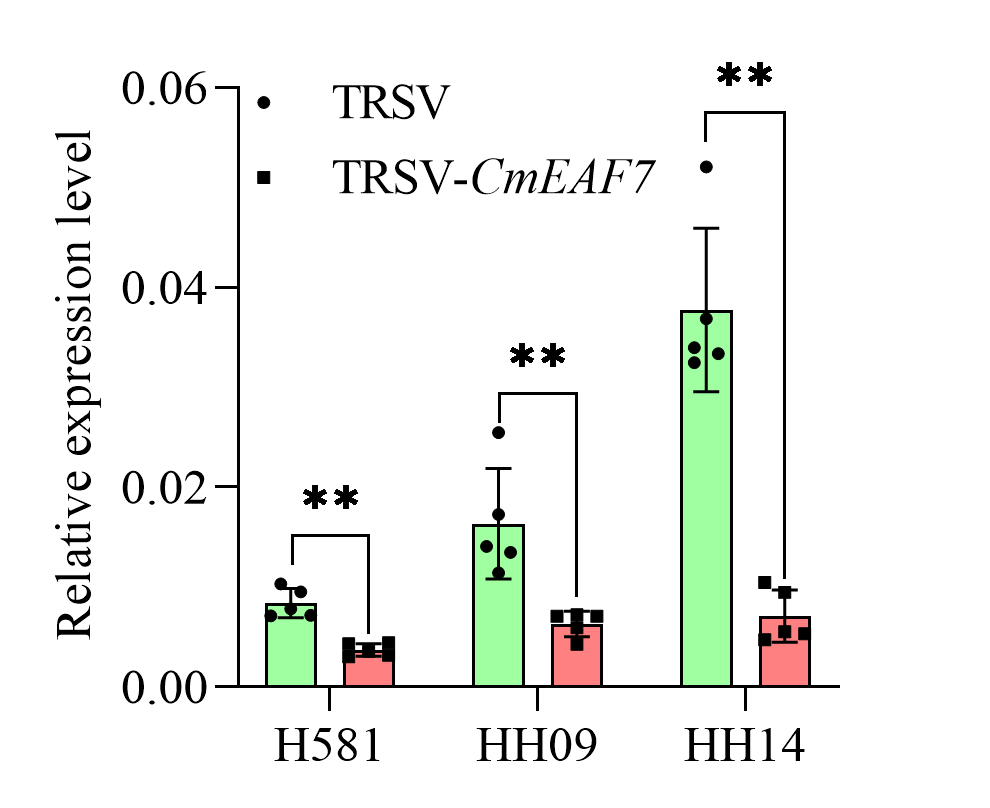


Figure S3

Figure S3. The expression levels of *CmEAF7* in H581, HH09, and HH14 VIGS seedlings. TRSV: Empty vector; TRSV-*CmEAF7*: VIGS vector for silencing of *CmEAF7* expression.


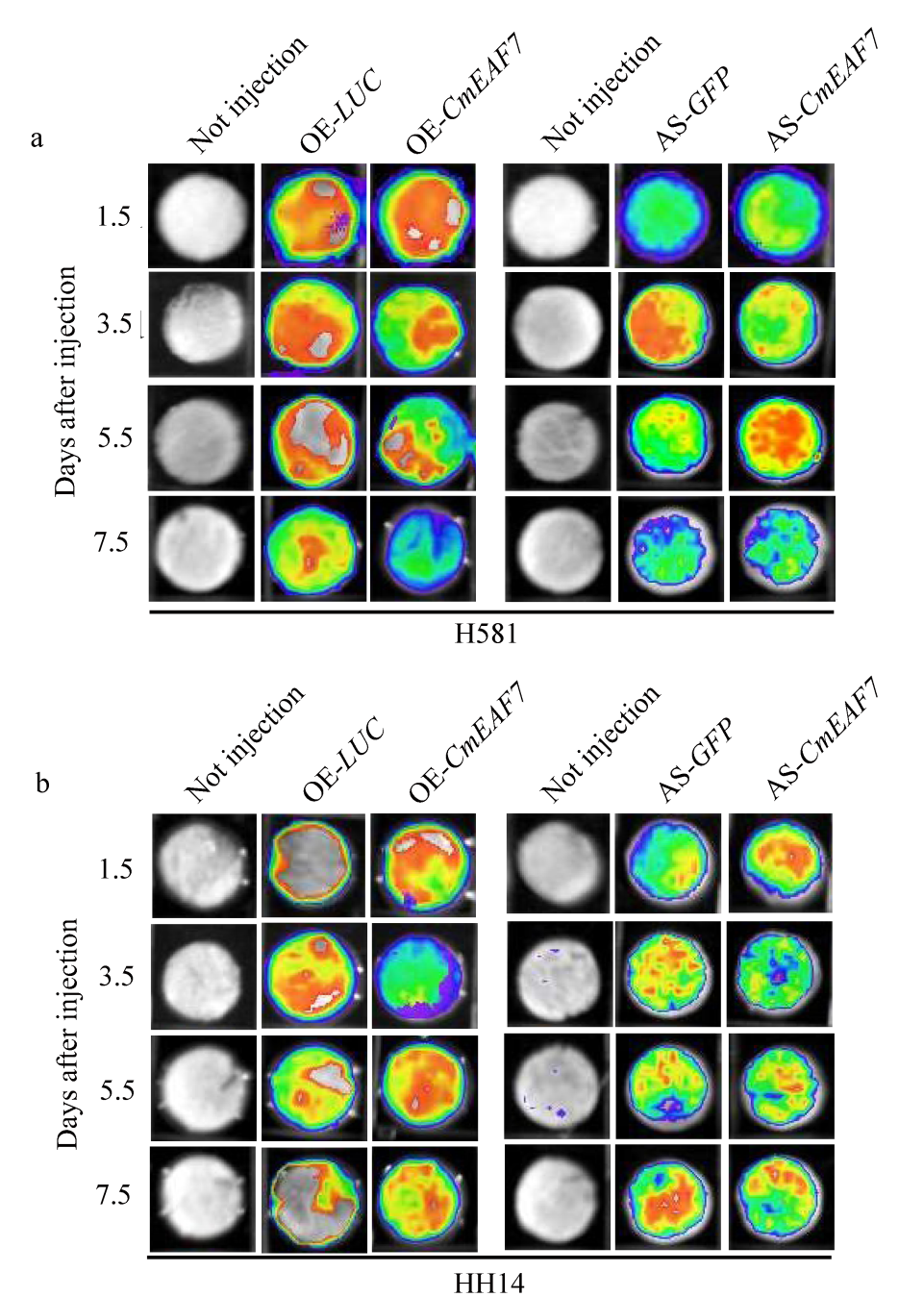


Figure S4

Figure S4. The LUC and GFP fluorescence signal intensities of samples after *CmEAF7* transient overexpression and silencing at different days after injection of H581 and HH14 fruits. (a): H581; (b): HH14.
