# Supplementary material for: Identification of candidate genes that regulate the trade-off between seedling cold tolerance and fruit quality in melon (Cucumis melo L.)
Source: Hortic Res. 2023 May 9;10(7):uhad093. doi: 10.1093/hr/uhad093 (PMC10321389; doi:10.1093/hr/uhad093)
Supplement: Web_Material_uhad093 [file web_material_uhad093.zip › Supplementary Tables.docx]

| Inbred line Number | Seed bank number | Inbred line name | Source |
| --- | --- | --- | --- |
| H581 | ZTG00581(China) | PI-169329 | Turkey |
| H1037 | ZTG01037(China) | PI-136213 | Canada |
| H929 | ZTG00929(China) | PI-476338 | Britain |
| HH14 | ZTG00969(China) | PI-164852 | India |
| HH09 | ZTG00509(China) | Earls Fanaoito | Britain |
| HH36 | M66(America) | NSL 34600 | America |
| HH94 | M408(America) | PI 378059 | Japan |
| HH90 | M316(America) | PI 618848 | China |

Supplementary table 1. The eight inbred melon lines used in this study.

Supplementary table 2. Candidate trade-off genes with sequence variations between

H581 and HH09.

| Gene ID | Chr | Position | Reference | H581 | HH09 | Description of variants |
| --- | --- | --- | --- | --- | --- | --- |
| MELO3C000086.2 | chr00 | 8969895 | C | C | T | missense_variant; Moderate |
|  |  | 8969896 | T | T | C | missense_variant; Moderate |
|  |  | 8970016 | C | C | T | missense_variant; Moderate |
|  |  | 8970138 | T | T | C | missense_variant; Moderate |
| MELO3C016749.2 | chr07 | 2345940 | T | T | TTAAAAATA | 3'UTR_variant |
|  |  | 2345939 | T | T | TAAAAAA | 3'UTR_variant |
| MELO3C012147.2 | chr10 | 2361960 | GTCTC | G | GTCTC | splice_donor_variant; High |
| MELO3C026791.2 | chr10 | 10483201 | C | T | C | intron_variant; Moderate |
| MELO3C006997.2 | chr08 | 55421 | GA | G | GA | 3'UTR_variant |
|  |  | 56904 | T | G | T | missense_variant; Moderate |
|  |  | 56930 | A | AGTGGGT | A | disruptive_inframe_insertion; Moderate |
| MELO3C010343.2 | chr02 | 17442607 | T | C | T | missense_variant; Moderate |
|  |  | 17444157 | A | T | A | missense_variant; Moderate |
| MELO3C024337.2 | chr01 | 36132568 | C | C | A | 3'UTR_variant |

Supplementary table 3. The expression levels of genes involved in the key steps of ABA biosynthesis.

| Gene_ID | Gene  Name | Description | Arabidopsis homologous gene | H581_FPKM | | | HH09_FPKM | | |
| --- | --- | --- | --- | --- | --- | --- | --- | --- | --- |
|  |  |  |  | Repeat 1 | Repeat 2 | Repeat 3 | Repeat 1 | Repeat 2 | Repeat 3 |
| MELO3C020872.2 | *CmABA1* | Zeaxanthin epoxidase, chloroplastic | AT5G67030 | 8.71 | 8.85 | 10.93 | 12.42 | 11.31 | 20.07 |
| MELO3C005129.2 | *CmABA2* | Xanthoxin dehydrogenase | AT1G52340 | 0.54 | 0.10 | 0.14 | 0.65 | 0.00 | 1.24 |
| MELO3C019659.2 | *CmABA3* | Molybdenum cofactor sulfurase | AT1G16540 | 12.91 | 9.05 | 10.75 | 13.05 | 11.38 | 15.87 |
| MELO3C023805.2 | *CmABA4* | Protein ABA DEFICIENT 4, chloroplastic | AT1G67080 | 1.59 | 1.43 | 1.67 | 3.72 | 1.68 | 0.87 |
| MELO3C027057.2 | *CmNCED3* | 9-cis-epoxycarotenoid dioxygenase, chlorplastic | AT3G14440 | 10.95 | 10.14 | 41.76 | 49.17 | 15.37 | 55.22 |

Supplementary table 4. Oligonucleotide primers used for qRT-PCR and PCR in this study.

| Gene name | Forward primers (5’ to 3’) | Reverse primers (5’ to 3’) | Type |
| --- | --- | --- | --- |
| *CmABA1* | CGGAGGAAGAAAGTCAACCA | TCTCGAAAACCACCACATCA | Q-PCR |
| *CmABA2* | GGTGCAAAGGTTTGTTTCGT | CGAAGGTCGTCTTCCACAGT | Q-PCR |
| *CmABA3* | CATGTCTGCTGGGATGATTG | CTGCGAAATGGATTGACCTT | Q-PCR |
| *CmABA4* | ACAGGCGTACCAGCAATACC | TCTTGATCTTCCACCGCAAT | Q-PCR |
| *CmNCED3* | AAGCATTCATTGCCCAAGAC | AATCACCGGTAAACCCTTCC | Q-PCR |
| *CmEAF7* | GCGAAGAAGCTTTGATCGTC  TTGCTTACCACAAACCTTCTTCG | CTCATCATCTGGCTTCAGCA  CAAAAAGGGAACGACCCTACC | Q-PCR OE  Q-PCR AS |
| *CmActin* | ATTCTTGCATCTCTAAGTACCTTCC | CCAACTAAAGGGAAATAACTCACC | Q-PCR |
| *CmEAF7* | TGTTTTAAATGCCTTTACGTAAGAACAAGACGGCATTTCTG | AACACACAAAACACCTACGTAGCTTCAGCATCTCCAGGTTA | PCR pTRSV2 |
| *CmEAF7* | TCTGAGCTCTCTAGAATGGAAAGCGGCGGCAAAG | TTTGGCGTCTTCCATAGACTCTTCTTTGACGAAGAAG | PCR pCAMBIA3301 |
| *CmEAF7* | GCCCTTGCTCACCATGAATTCATGGAAAGCGGCGGCAAAG | TCTTCACTGTTGATACATATGAGACTCTTCTTTGACGAAGAAG | PCR pRI101 |

Note: These underlined sequences represent orthologous arms.
